# Functionalization of Photocrosslinkable GelMA Hydrogel With Metal Oxides for Direct Pulp Capping

**DOI:** 10.1155/bmri/5774660

**Published:** 2026-04-26

**Authors:** Ester Alves Ferreira Bordini, Ligia Espoliar Corrêa, Fernanda Balestrero Cassiano, Érika Soares Bronze-Uhle, Isabela Sanches Pompeo da Silva, Camila Correa da Silva Braga de Melo, Carlos Alberto de Souza Costa, Diana Gabriela Soares

**Affiliations:** ^1^ Department of Dental Materials and Prosthodontics, Ribeirão Preto School of Dentistry, University of São Paulo–USP, Ribeirão Preto, São Paulo, Brazil, usp.br; ^2^ Department of Operative Dentistry, Endodontics, And Dental Materials, Bauru School of Dentistry, University of São Paulo–USP, Bauru, São Paulo, Brazil, usp.br; ^3^ Department of Physiology and Pathology, Araraquara School of Dentistry, University Estadual Paulista–UNESP, Araraquara, São Paulo, Brazil

**Keywords:** direct pulp capping, GelMA, hydrogel, metal oxides, mineralized matrix, stem cells from apical papilla

## Abstract

In this study, a photocrosslinkable methacrylated gelatin (GelMA) hydrogel, functionalized with metal oxide nanoparticles, was developed to support dentin regeneration in direct pulp capping applications. Magnesium (MgO), silicon (SiO_2_), strontium (SrO), and zinc (ZnO) were incorporated into GelMA at concentrations of 0.1%, 0.2%, 0.3%, 0.5%, and 1% (v/v) to assess cytotoxicity in human stem cells from the apical papilla (SCAPs). Based on the results, MgO, SiO_2_, and SrO were selected for further functionalization of GelMA at reduced concentrations (0.1%, 0.075%, 0.05%, and 0.025%; v/v) to evaluate the odontogenic potential of the formulations. Physicochemical analyses were performed to characterize the biomaterials. ZnO exhibited cytotoxicity at all tested concentrations. In contrast, the other oxides demonstrated excellent bioactivity and enhanced cell proliferation, except for SrO 0.025%. A significant increase in alkaline phosphatase (ALP) activity and mineralized matrix deposition was observed across all groups, with the most pronounced effects seen in MgO 0.05%, MgO 0.025%, and SrO 0.075%. The functionalized hydrogels exhibited a porous microstructure with a slow and controlled release of oxide‐derived species over 14 days. FTIR analysis confirmed the complexation of Mg^2+^, Si^4+^, and Sr^2+^ ions within the GelMA structure. In conclusion, our results demonstrate that GelMA functionalized with MgO 0.05%, SiO_2_, and SrO 0.075% enhances odontoblastic differentiation and promotes reparative mineralized matrix deposition under osteogenic conditions, favoring reparative dentinogenesis. These findings highlight the promising potential of this biomaterial for future applications in direct pulp capping procedures.

## 1. Introduction

Regenerating mineralized tissues such as dentin remains a considerable clinical challenge in dentistry. For successful regeneration that promotes homeostasis and preserves pulp tissue vitality, the biomaterials employed must not only exhibit excellent biocompatibility, but also conform precisely to the defect site, adhere effectively to the surrounding tissues, and facilitate the formation of a mineralized barrier resembling native dentin [1, 2]. Such a barrier is critical to ensuring the long‐term success of the treatment by preventing microleakage and reducing the risk of bacterial infection or reinfection [3].

The development of a biomimetic regenerative environment capable of providing appropriate signaling cues to stimulate the migration, proliferation, and differentiation of mesenchymal pulp cells can be achieved through the use of functional biomaterials [4]. The selection of natural polymers for formulating these materials represents a promising approach, as they offer a structural framework that closely mimics the extracellular matrix (ECM) of dentin, an ECM rich in glycosaminoglycans and essential for supporting and regulating cellular metabolism [5]. Gelatin, a soluble protein derived from the partial hydrolysis of collagen, is an attractive candidate due to its low cost, favorable hydrophilicity, and excellent biocompatibility, attributes largely conferred by the presence of the RGD (arginine–glycine–aspartate) sequence. These characteristics render the surface of this polymer highly conducive to cell adhesion, proliferation, and differentiation, while also enabling effective biochemical signaling necessary for tissue regeneration [6, 7].

Despite its numerous advantages, gelatin exhibits low‐mechanical stability and rapid degradation under physiological conditions [6]. To overcome these limitations, chemical modification through the incorporation of methacrylate groups enables the formation of a stable, photosensitive, biocompatible, and biodegradable hydrogel with broad clinical potential, particularly in settings where minimally invasive restorative and regenerative therapies are desired [8, 9]. However, several studies have reported that the regenerative capacity of methacrylated gelatin (GelMA) in promoting mineralized tissue regeneration remains limited when used in its unmodified and nonfunctionalized form [10–13].

One approach to accelerate tissue repair and regeneration, as well as to promote the formation of a functional mineralized dentin barrier, is the functionalization of hydrogels with bioactive compounds. These biomaterials not only enable the sustained and controlled release of low doses of incorporated drugs but also facilitate the uniform distribution of both the hydrogel matrix and bioactive molecules within the target site [14]. This strategy enhances the recruitment of human dental pulp progenitor cells to the defect area, thereby inducing odontogenic differentiation by mimicking the intrinsic repair mechanisms of pulp tissue [5, 15].

The incorporation of inorganic nanoparticles into injectable hydrogels holds considerable promise due to their stability, low immunogenic potential, cost‐effectiveness, and pleiotropic effects on regenerative cellular activity [16–18]. Magnesium (MgO) and zinc ZnO) play crucial roles in physiological and metabolic cellular processes by directly influencing cell proliferation and osteo/odontogenic differentiation, thereby promoting significant mineralized tissue deposition both in vitro and in vivo [19, 20]. Additionally, the incorporation of strontium (SrO) into various polymer matrices facilitates the formation of oxygenated functional groups, which enhance biocompatibility and osteo/odontogenic potential, while promoting efficient gas and metabolic exchange within the biomaterial network [16, 21].

Silicon (SiO_2_) exerts its effects by binding to glycosaminoglycans, thereby enhancing cell proliferation and upregulating the expression of osteogenesis‐related genes, which stimulates the formation of mineralized tissue with appropriate quality and density [22, 23]. It has been demonstrated that both SrO and SiO_2_ primarily act during the early stages of mineralization. Furthermore, they promote hydroxyapatite precipitation and participate in the activation of macrophage and osteoclast pathways, contributing to the regulation of bone resorption and remodeling [21, 23].

In this context, the present study developed an injectable GelMA hydrogel, capable of in situ light curing, combined with metal oxides (magnesium, strontium, silicon, and zinc) to enhance dentin regeneration aimed at clinical applications of direct pulp capping (DPC). Accordingly, this work was designed as an early‐stage biological and material screening aimed at assessing the bioactivity and odontogenic differentiation potential of metal oxide–functionalized GelMA hydrogels prior to further translational evaluation. The biomaterials were thoroughly characterized with respect to their physicochemical properties, sustained release of inorganic ions, and their ability to induce odontogenic differentiation in vitro upon interaction with human stem cells from the apical papilla (SCAPs). This cell type was chosen for this study due to its high proliferative capacity, migratory potential, and odontogenic differentiation, which together contribute to the formation of a robust and homogeneous mineralized barrier [24, 25].

## 2. Materials and Methods

### 2.1. Preparation and Dispersion Characterization of Metal Oxides

Nanoparticles of MgO (≤ 50 nm, Sigma‐Aldrich, St. Louis, Missouri, United States), SiO_2_ (5–20 nm, Sigma‐Aldrich), SrO (99.9% trace metals basis, Sigma‐Aldrich), and ZnO (< 100 nm, Sigma‐Aldrich) were dispersed at a final concentration of 5% (w/v) in two different vehicles: ultrapure water (upH_2_O) and a solvent consisting of bovine serum albumin (970‐*μ*L BSA; 0.05*%*w/v) combined with anhydrous ethanol (30‐*μ*L EtOH) [26]. The solutions were continuously stirred for 1 min and subsequently spread onto glass slides, which were then covered with coverslips to assess the dispersive capacity of the metal oxide particles. For each oxide, three representative images were captured using an optical microscope at 20× magnification (EVOS FLoid Cell Imaging Station; Invitrogen, Carlsbad, California, United States). To evaluate the influence of the different vehicles on particle dispersion, particle and/or agglomerate sizes were measured using ImageJ software (National Institutes of Health, Bethesda, Maryland, United States).

### 2.2. Functionalization of GelMA Hydrogel With Metal Oxides

The GelMA hydrogel was synthesized following the protocol described by da Silva et al. Briefly, 0.075% (w/v) of the photoinitiator lithium phenyl‐2,4,6‐trimethylbenzoylphosphinate (LAP; Sigma‐Aldrich) was first dissolved in phosphate‐buffered saline (PBS) (1×; Fisher Scientific, Hampton, New Hampshire, United States), sterilized through a 0.22‐*μ*m filter, and subsequently added to the GelMA solution at a final concentration of 15% (w/v). To maintain aseptic conditions, the freeze‐dried GelMA was presterilized by exposure to ultraviolet (UV) light for 30 min prior to preparation.

Working solutions at 5% (w/v) were prepared by dissolving the metal oxide nanoparticles in the BSA/EtOH solvent. The initial concentration range was selected based on previous studies reporting the bioactive potential of metal oxides on undifferentiated mesenchymal stem cells [27–32].

Subsequently, a pilot study was conducted to refine these concentrations and determine the optimal working range. Accordingly, the metal oxides were individually incorporated into the GelMA solution at final concentrations of 0.1%, 0.2%, 0.3%, 0.5%, and 1% (v/v) as part of a pilot study designed to assess cytocompatibility. In this context, the reported v/v values refer exclusively to the volume fraction of the 5% (w/v) metal oxide stock suspension added to the liquid GelMA hydrogel prior to photopolymerization.

Each homogenized hydrogel formulation was dispensed into 96‐well plates (100‐*μ*L/well) and photocrosslinked for 30 s using a Bluephase N light source (1200 mW/cm^2^; Ivoclar Vivadent, Barueri, São Paulo, Brazil). The resulting specimens (5 − mm diameter × 2 − mm thickness) were evaluated for cell viability (n = 2). Cells cultured (1 × 10^4^ cells/hydrogel) beneath hydrogels without incorporated oxides or solvents were used as negative controls. Additionally, the cytotoxicity of the BSA/EtOH solvent was assessed by exposing SCAPs to GelMA samples containing 1% solvent in direct contact for 24 h.

Based on the preliminary results, which indicated increased cytotoxicity at higher concentrations, lower concentrations (0.025*%*–0.1*%*v/v) were selected for subsequent biological assays. The corresponding final mass concentrations of MgO, SiO_2_, and SrO incorporated into the GelMA hydrogels are summarized in Table 1.

**Table 1:** Final mass concentrations (mg/mL and wt%) of MgO, SiO_2_, SrO, and ZnO incorporated into GelMA hydrogel (100 *μ*L), calculated based on the volume fraction (v/v) of a 5% (w/v) metal oxide stock suspension added prior to photopolymerization.

Experimental stage	Metal oxides	*v*/*v*(%)	Final concentration (mg/mL)	Final concentration (wt%)
Pilot study	MgO SiO_2_ SrO ZnO	0.1	0.05	0.005
		0.2	0.1	0.01
		0.3	0.15	0.015
		0.5	0.25	0.025
		1.0	0.5	0.05
Biological assays	MgO SiO_2_ SrO	0.025	0.0125	0.00125
		0.05	0.025	0.0025
		0.075	0.0375	0.00375
		0.1	0.05	0.005

### 2.3. Biological Analyses

#### 2.3.1. Cell Culture and Viability Assay

Prior to the biological analyses, a primary culture of SCAPs was established via enzymatic digestion of apical papilla tissue obtained from two human third molars with incomplete root formation, collected from healthy male donors aged 18 and 19 years. Cells obtained from both donors were pooled after primary isolation to generate a single, mixed SCAP population, which was used consistently across all experiments and experimental groups. Written informed consent was obtained from all donors (Protocol No. 26153119.6.0000.5417), and the study was approved by the Ethics Committee of the Bauru School of Dentistry, Bauru, São Paulo, Brazil. All experiments were performed using SCAPs at Passage 4.

Cells were cultured in complete *α*‐MEM medium (Minimum Essential Medium Eagle Alpha; Gibco, Invitrogen, Carlsbad, California, United States), supplemented with 10% fetal bovine serum (FBS), L‐glutamine, and 1% penicillin‐streptomycin (Gibco). Cells were maintained at 37°C in a humidified atmosphere containing 5% CO_2_. Mycoplasma contamination was routinely assessed using the MycoFluor Mycoplasma Detection Kit (Molecular Probes, Eugene, Oregon, United States), and no evidence of contamination was detected throughout the study.

Upon reaching approximately 80% confluence, cells were trypsinized using 0.25% trypsin‐EDTA (Gibco) and seeded onto GelMA hydrogels functionalized with metal oxides at a density of 1 × 10^4^ cells per hydrogel. The cultures were incubated overnight to allow cell attachment and proliferation. Subsequently, live/dead staining (Live/Dead Cell Viability/Cytotoxicity Kit, Invitrogen) was performed to assess viable (green) and nonviable (red) cells on the hydrogel surfaces. For this assay, the culture medium was replaced with 200 *μ*L of *α*‐MEM without FBS, supplemented with calcein AM (2 *μ*M) and ethidium homodimer‐1 (4 *μ*M). Samples were incubated for 30 min before fluorescence images were captured using an EVOS FLoid Cell Imaging Station (Invitrogen).

#### 2.3.2. Assessment of Cell Proliferation

Following the assessment of cell viability, a serial dilution was performed to incorporate MgO, SiO_2_, and SrO into GelMA hydrogels at concentrations of 0.025%, 0.05%, 0.075%, and 0.1% (v/v), as described above. SCAPs were seeded onto the developed formulations at a density of 1 × 10^4^ cells per hydrogel and incubated for up to 14 days in complete *α*‐MEM medium, with the culture medium refreshed every 2 days. At predetermined time points, the cell/hydrogel constructs (n = 6) were incubated for 4 h at 37°C and 5% CO_2_ in *α*‐MEM containing Alamar blue reagent (10:1 dilution; Invitrogen). Cell proliferation was quantified by measuring fluorescence intensity (excitation at 540 nm and emission at 590 nm) using a Synergy H1 microplate reader (BioTek, Winooski, United States). Cells cultured on pure GelMA hydrogels were set as the reference for 100% proliferation, and proliferation rates for other groups were calculated relative to this control.

#### 2.3.3. Evaluation of Odontogenic Differentiation Induced by Metal Oxide–Functionalized Hydrogels

At this stage, the hydrogel/SCAP constructs (1 × 10^4^ cells/hydrogel) were cultured in osteogenic medium (complete *α*‐MEM supplemented with 50 *μ*g/mL ascorbic acid and 5‐mM *β*‐glycerophosphate; Sigma‐Aldrich) to evaluate the odontogenic potential of the hydrogel formulations containing metal oxides. After 7 days of incubation, alkaline phosphatase (ALP) activity was assessed (n = 8) using an endpoint assay kit (Labtest Diagnóstico S.A., Lagoa Santa, Minas Gerais, Brazil). Samples underwent cell lysis by incubation in 0.1% sodium lauryl sulfate solution (Sigma‐Aldrich) for 40 min. The resulting supernatant was collected, and half of the samples were incubated with thymolphthalein monophosphate solution (22 mmol/L, pH 10.1) at 37°C for 15 min for ALP quantification via spectrophotometry (590 nm; Synergy H1, BioTek). The remaining half of the samples were used for total protein quantification by incubation with Lowry/Folin–Ciocalteau reagent (Sigma‐Aldrich) for 30 min, followed by absorbance reading at 655 nm. ALP activity was normalized to total protein content. The control group (pure GelMA) was defined as 100% ALP expression.

Mineralized matrix deposition by SCAPs seeded on hydrogels functionalized with metal oxides (1 × 10^4^ cells/hydrogel) was quantified after 14 days of culture in osteogenic medium. The cell/hydrogel constructs (n = 8) were fixed at 4°C in 70% ethanol for 1 h, followed by incubation with 40‐mM alizarin red solution (pH 4.2; Sigma‐Aldrich) for 15 min. Excess dye was removed by successive washes with deionized water, and mineralized nodules were subsequently solubilized using 10‐mM cetylpyridinium chloride solution (pH 7.0; Sigma‐Aldrich). Absorbance was measured at 560 nm (Synergy H1, BioTek). Cells cultured on pure GelMA hydrogels were used as the reference control and considered to represent 100% mineralized matrix deposition. Hydrogels functionalized with metal oxides but without cells were used as background controls (n = 8).

#### 2.3.4. Cell Adhesion and Spreading Assay

Cell adhesion and spreading were evaluated by staining actin filaments. Cells cultured on the hydrogels (n = 2; 1 × 10^4^ cells/hydrogel) were fixed with 4% paraformaldehyde (PFA) (Sigma‐Aldrich), permeabilized using 0.1% Triton X‐100 (Thermo Fisher Scientific, Waltham, Massachusetts), and incubated with Alexa Fluor 488 Phalloidin (1:50 dilution; Life Technologies, Carlsbad, California) for 30 min. Nuclear counterstaining was performed with DAPI (ProLong, Thermo Fisher Scientific) following the manufacturer′s instructions. Fluorescence microscopy (EVOS Floid, Invitrogen) was used to capture representative images of the hydrogel surfaces.

### 2.4. Physicochemical Characterization of Functionalized GelMA Hydrogels

#### 2.4.1. Morphology and Surface Topography

The various hydrogel formulations were characterized using scanning electron microscopy (SEM) (JEOL JSM‐6610LV, Peabody, Massachusetts, United States) operated at an accelerating voltage of 10–15 kV. Samples functionalized with MgO (0.025%, 0.05%), SiO_2_ (0.025%, 0.05%), and SrO (0.075%, 0.1%) were freeze‐dried at −52°C, mounted on metal stubs, and sputter‐coated with gold. SEM images were acquired at 250× and 500× magnifications (n = 4) to assess the morphology and surface topography of the hydrogels.

#### 2.4.2. Degree of Porosity and Pore Diameter

Porosity and pore diameter were quantified from SEM images captured at 250× magnification using ImageJ software (National Institutes of Health). Pore diameter measurements were performed using the straight‐line tool. Porosity percentage was calculated by applying the threshold tool to the SEM images, which measures the ratio of pore area relative to the total image area [33]. For each sample (n = 4), 25 pores were randomly selected to calculate an average pore diameter.

#### 2.4.3. Swelling Behavior and Degradation Assessment

In order to assess the influence of metal oxide functionalization on hydrogel degradability, samples (n = 6) were incubated in PBS (pH 7.0) with or without collagenase Type I enzyme (1 U/mL; Sigma‐Aldrich). After reaching maximum swelling (24 h at 37°C), the initial wet weight of each hydrogel was measured using a high‐precision balance (Mettler Toledo XS105 DualRange; Columbus, Ohio, United States). Samples were maintained immersed in PBS at 37°C throughout the experiment period. At predetermined time points (1, 3, 7, 14, 21, and 28 days), the wet weight was recorded after gently blotting excess surface moisture with absorbent paper. For each experimental group, the mean wet weight at Day 0 was defined as 100%, and the wet weights at subsequent time points were normalized to this baseline. Hydrogel degradation was expressed as the relative mass loss over time and reported as a percentage decrease in wet weight compared with the initial value.

Samples (n = 5) were immersed in PBS (pH 7.0) at 37°C for 24 h to reach maximum swelling. The initial wet weight (Pi) was then measured using an analytical balance (Mettler Toledo XS105 DualRange; Columbus, Ohio, United States). After incubation in PBS at 37°C for 6, 12, and 24 h, samples were gradually frozen (−20°C overnight, then −80°C for 24 h) and subsequently freeze‐dried (−52°C for 24 h) to obtain the dry weight (Ps) of the hydrogels. The degree of swelling (%) was calculated using the formula: Gi (*%*) = (Pi − Ps)/Ps × 100 [34].

#### 2.4.4. Apparent Metal Oxide–Derived Species Release

The apparent release of metal oxide–derived species from GelMA hydrogels was evaluated using UV‐Vis spectroscopy (Synergy H1) at a specific wavelength of 600 nm for Mg^2+^, 235 nm for Si^4+^, and 235 nm for Sr^2+^. Samples (n = 6) were incubated at 37°C in Eppendorf tubes containing 1 mL of PBS (pH 7.0). At predetermined time points (3, 15 h and 1, 3, 7, and 14 days), the tubes were mixed thoroughly for 30 s, and 200 *μ*L of the supernatant was collected and stored at −20°C until analysis. Fresh PBS was then added to each tube to restore the initial volume. The apparent cumulative release was quantified over time and expressed as concentration (*μ*g/*μ*L), based on metal oxide–specific standard curves prepared in PBS, which was used as the primary blank (Figure S1). Pure GelMA hydrogels without metal oxides, incubated in PBS, were analyzed in parallel with plain PBS for up to 14 days to evaluate potential interference of GelMA‐derived components with absorbance at the selected wavelengths (600 and 235 nm). Since significant differences were detected from Day 3 onward (Figure S2), GelMA + PBS samples were subsequently used as blanks to correct for background absorbance in samples containing metal oxides.

#### 2.4.5. Fourier‐Transform Infrared Spectroscopy (FTIR)–ATR Analysis

Chemical modifications in the GelMA structure induced by the incorporation of MgO (0.025% and 0.05%), SiO_2_ (0.025% and 0.05%), and SrO (0.075% and 0.1%) were analyzed using FTIR. Spectra were acquired using a PRESTIGE‐21 FTIR spectrometer (Shimadzu, Japan) equipped with an attenuated total reflectance (ATR) accessory. For all hydrogel formulations, FTIR spectra were recorded in ATR mode with 32 scans, a resolution of 4 cm^−1^, over the wavenumber range of 450–4000 cm^−1^.

#### 2.4.6. Statistical Analysis

All experiments were independently performed in triplicate. For all assays, the reported n values correspond to the number of independently prepared and cultured GelMA hydrogel samples per group, which were considered biological replicates and used as the experimental units for statistical analysis. Technical replicates, when applicable, were averaged and not treated as independent observations.

Statistical analyses were conducted using GraphPad Prism 10 (GraphPad Software Inc., Boston, Massachusetts, United States). Data are presented as mean ± standard deviation (SD). Differences between groups were evaluated using one‐way or two‐way analysis of variance (ANOVA), as appropriate, followed by Tukey′s post hoc test. For the characterization of metal oxide dispersion in solvents, data were analyzed using the nonparametric Kruskal–Wallis test followed by Dunn′s post hoc test, due to the non‐normal distribution of the data. A p value of less than 0.05 was considered statistically significant. For each assay, the appropriate control groups described in the Methods section (including pure GelMA hydrogels, solvent controls when applicable, and assay‐specific background controls) were included in the corresponding statistical comparisons.

## 3. Results

### 3.1. Characterization of Metal Oxide Dispersion in Solvents

**Figure 1:**
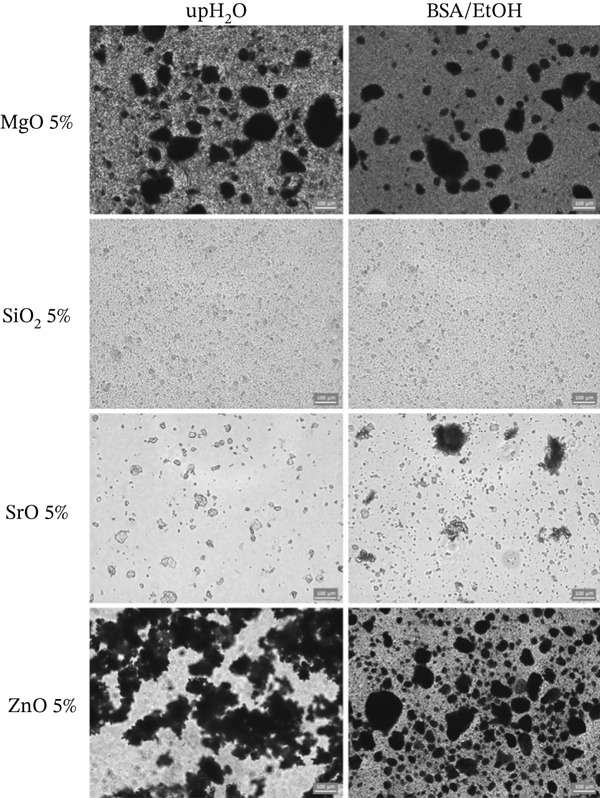
Representative micrographs of metal oxide particles dispersed in ultrapure water (upH_2_O) and BSA/ethanol (BSA/EtOH) solvent, acquired via digital microscopy under white light illumination at 20× magnification (scale bar: 100 *μ*m).

The use of the BSA/ethanol (BSA/ETOH) solvent resulted in a more uniform dispersion of SrO and ZnO nanoparticles on the glass slide surface compared with ultrapure water, although some particle agglomerates were still observed (Figure 1). Moreover, this solvent significantly reduced the average particle size from 27.97 and 159.67 *μ*m to 16.88 and 25.72 *μ*m, respectively (Table 2; p < 0.05). In contrast, no statistically significant differences in particle size were observed for MgO and SiO_2_ between the two dispersion vehicles (Table 2; p > 0.05), indicating that solvent composition did not significantly affect the dispersion behavior of these oxides under the conditions evaluated. Representative micrographs corroborate these findings, showing comparable particle distributions for MgO and SiO_2_ in both ultrapure water and BSA/ETOH (Figure 1).

**Table 2:** Particle diameters of MgO, SiO_2_, SrO, and ZnO measured in ultrapure water (upH_2_O) and BSA/ethanol (BSA/EtOH) solvent.

		MgO	SiO_2_	SrO	ZnO
upH_2_O	m (SD)	43.64 (59.5) a	17.76 (12.4) a	27.97 (17.7) a	159.67 (145.9) a
	min	3.61	6.71	9.06	7.62
	max	231.87	57.39	92.35	448.88
BSA/EtOH	m (SD)	27.48 (42.4) a	13.36 (7.9) a	16.88 (11.9) b	25.72 (29.7) b
	min	2.83	5.66	4.47	5.00
	max	185.16	38.83	50.01	191.64

*Note:* min: smallest particle diameter; max: largest particle diameter. Different lowercase letters indicate statistically significant differences within the same group for the different solvents evaluated (Kruskal–Wallis test with Dunn′s post hoc test; p < 0.05).

Abbreviations: m, mean; SD, standard deviation.

### 3.2. Screening of Different Metal Oxide Concentrations in GelMA Hydrogel

**Figure 2:**
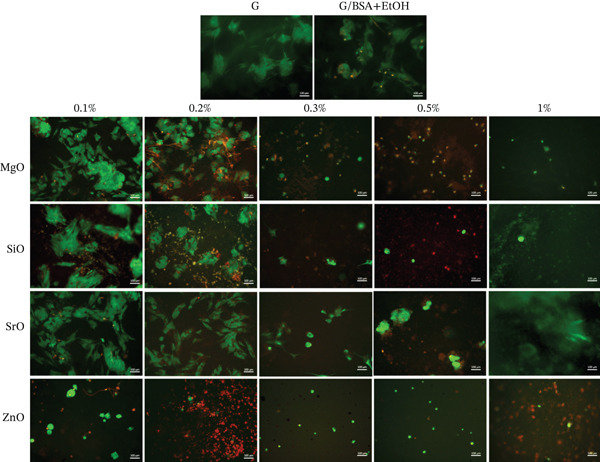
Live/dead assay. Representative images (20×) of SCAPs seeded on the surface of hydrogels functionalized with MgO, SiO_2_, SrO, and ZnO at concentrations of 0.1%, 0.2%, 0.3%, 0.5%, and 1%. Green indicates viable cells; red indicates dead cells (scale bar: 100 *μ*m).

In Figure 2, live/dead staining demonstrated that GelMA containing 1% BSA/ETOH solvent induced minimal cell death compared with pure GelMA (control). GelMA formulations incorporating MgO, SiO_2_, and SrO together with the dispersing agent exhibited predominantly viable cells distributed across the sample surfaces at concentrations of 0.2% and 0.1%. Conversely, higher concentrations of MgO, SiO_2_, and SrO (0.3%, 0.5%, and 1%) were associated with an increased proportion of dead cells, indicating concentration‐dependent cytotoxic effects on SCAPs. ZnO exhibited pronounced cytotoxicity at all tested concentrations, even after predispersion, and was therefore excluded from subsequent experiments. The dispersion method employed improved nanoparticle homogenization and was compatible with cell viability within a defined concentration range.

### 3.3. Biological Characterization

#### 3.3.1. Cell Viability and Proliferation Analysis

**Figure 3:**
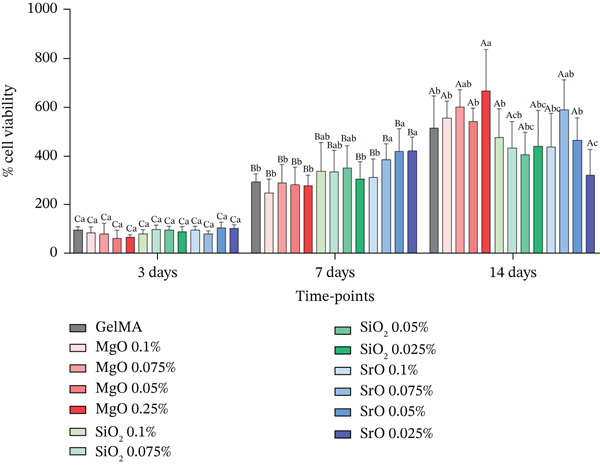
Bar graph showing mean values and standard deviations obtained at each analysis period using the Alamar blue assay (*n* = 6). Different capital letters indicate statistically significant differences between analysis periods within each group; different lowercase letters indicate statistically significant differences between groups at the same analysis period (two‐way ANOVA/Tukey, *p* < 0.05).

**Figure 4:**
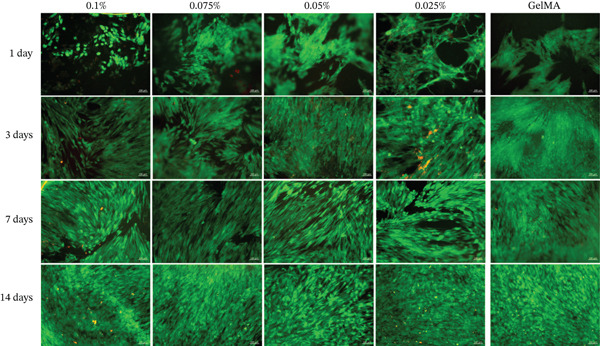
Live/dead assay. Representative images (20×) of hydrogels functionalized with varying concentrations (0.1%, 0.075%, 0.05%, and 0.025%) of MgO. *G* *r* *e* *e* *n* = *v* *i* *a* *b* *l* *e* *c* *e* *l* *l* *s*; *r* *e* *d* = *d* *e* *a* *d* *c* *e* *l* *l* *s* (scale bar: 100 *μ*m).

**Figure 5:**
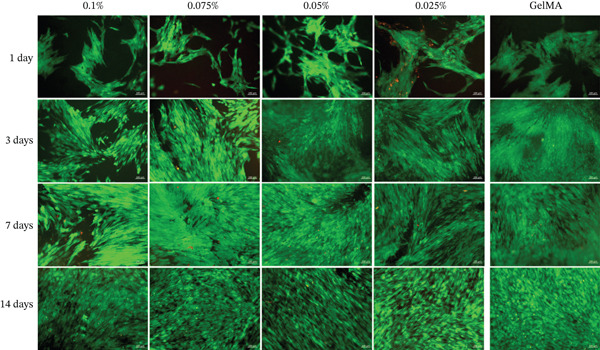
Live/dead assay. Representative images (20×) of hydrogels functionalized with different concentrations (0.1%, 0.075%, 0.05%, and 0.025%) of SiO_2_. *G* *r* *e* *e* *n* = *v* *i* *a* *b* *l* *e* *c* *e* *l* *l* *s*; *r* *e* *d* = *d* *e* *a* *d* *c* *e* *l* *l* *s* (scale bar: 100 *μ*m).

**Figure 6:**
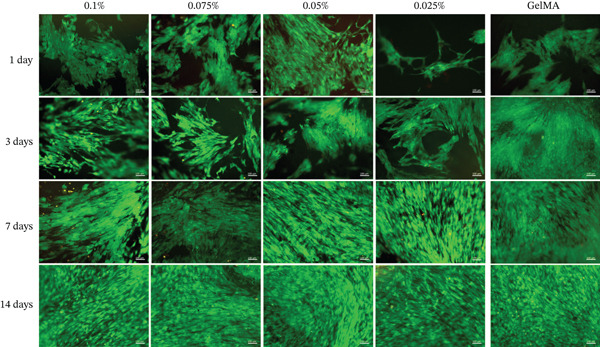
Live/dead assay. Representative images (20×) of hydrogels functionalized with different concentrations (0.1%, 0.075%, 0.05%, and 0.025%) of SrO. *G* *r* *e* *e* *n* = *v* *i* *a* *b* *l* *e* *c* *e* *l* *l* *s*; *r* *e* *d* = *d* *e* *a* *d* *c* *e* *l* *l* *s* (scale bar: 100 *μ*m).

Alamar blue assay results are presented in Figure 3. All tested GelMA formulations functionalized with metal oxides demonstrated bioactivity, promoting cell proliferation throughout the observation period. Notably, SCAPs seeded on GelMA functionalized with MgO exhibited increased proliferation at 14 days across all concentrations tested. Conversely, all SiO_2_ formulations showed lower proliferation rates at 14 days (430%–490%) compared with pure GelMA (560%). Additionally, the SrO 0.025% formulation showed a reduction in cell proliferation at 14 days (324.0%) compared with 7 days (423.3%). Live/dead staining confirmed the presence of predominantly viable cells on the surface of hydrogels containing MgO (Figure 4), SiO_2_ (Figure 5), and SrO (Figure 6) at all tested concentrations and time points. Although a small number of dead cells (red fluorescence) were observed across groups, overall cell viability was not compromised.

#### 3.3.2. Metal Oxide–Functionalized GelMA Enhances Cell Differentiation

After 7 days, SCAPs cultured in close contact with GelMA hydrogels functionalized with MgO at 0.05% and 0.025%, as well as SiO_2_ at all tested concentrations, exhibited a slight increase in ALP activity; however, no statistically significant differences were observed compared with the control group (pure GelMA). Hydrogels containing SrO at concentrations of 0.1%, 0.075%, and 0.05% demonstrated, on average, a 30% increase in ALP expression, whereas the 0.025% SrO group showed a 13% reduction in ALP activity (Figure 7a).

Figure 7(a) Bar graph showing mean values and standard deviations for the percentage of ALP activity, (b) bar graph showing mean values and standard deviations for mineral deposition as measured by the alizarin red assay, and (c) representative images of GelMA samples containing metal oxides, with and without cells (background control), stained with alizarin red. Different letters indicate statistically significant differences (one‐way ANOVA/Tukey; *p* < 0.05; *n* = 8).

**Figure:**
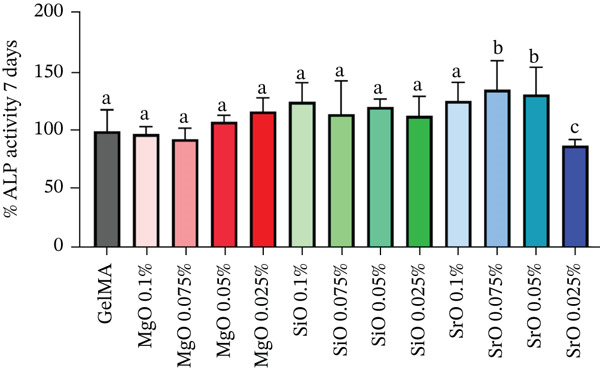
(a)

**Figure:**
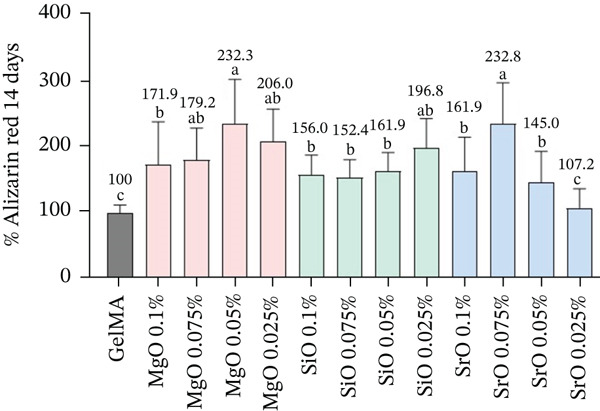
(b)

**Figure:**
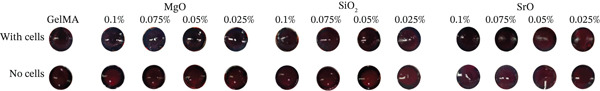
(c)

Alizarin red analysis (Figure 7b) demonstrated a statistically significant increase in mineralized matrix deposition in all metal oxide–functionalized hydrogels relative to the control, with the exception of the 0.025% SrO group. Notably, SCAPs cultured on GelMA functionalized with MgO at 0.05% and 0.025%, as well as SrO at 0.075%, exhibited the highest levels of mineral deposition, exceeding 200% relative to the control (232.3%, 206%, and 232.8%, respectively). Additionally, SiO_2_ at 0.05% and 0.025%, along with SrO at 0.1%, showed a significant but comparatively lower increase in mineral deposition (161.9%, 196.8%, and 161.9%, respectively). Representative images of alizarin red stained hydrogels without cells, with or without oxide particles (Figure 7c) confirmed a degree of nonspecific dye adsorption by the biomaterial matrix. Nevertheless, groups exhibiting higher quantitative mineral deposition showed more intense surface staining, supporting the bioactive and osteo/odontogenic potential of these formulations under osteogenic conditions.

Based on these findings, GelMA hydrogels functionalized with MgO (0.05% and 0.025%), SiO_2_ (0.05% and 0.025%), and SrO (0.1% and 0.075%) were selected for further analyses.

#### 3.3.3. Cell Adhesion and Spreading on Metal Oxide–Functionalized GelMA Hydrogels

**Figure 8:**
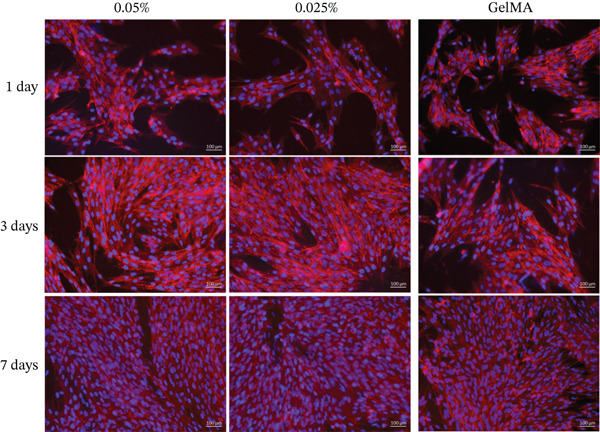
Cell adhesion and spreading assay. Representative images (20×) of the surface of GelMA hydrogels containing different concentrations (0.1%, 0.075%, 0.05%, and 0.025%) of magnesium oxide (MgO). Blue staining indicates cell nuclei; red staining indicates F‐actin filaments (scale bar: 100 *μ*m).

**Figure 9:**
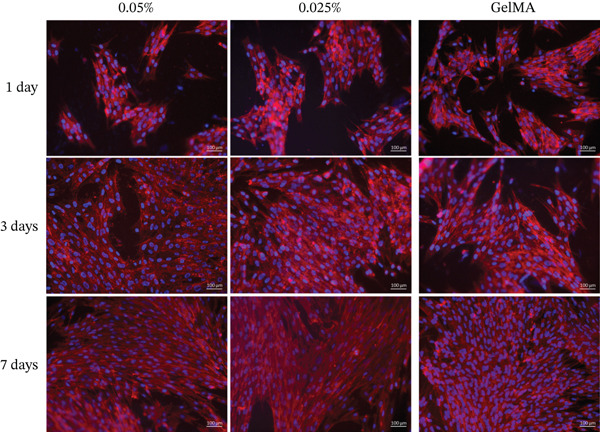
Cell adhesion and spreading assay. Representative images (20×) of the surface of GelMA hydrogels containing different concentrations (0.1%, 0.075%, 0.05%, and 0.025%) of silicon dioxide (SiO_2_). Blue staining indicates cell nuclei; red staining indicates F‐actin filaments (scale bar: 100 *μ*m).

**Figure 10:**
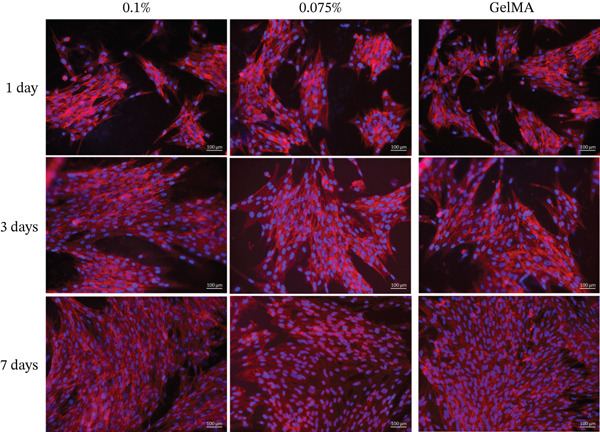
Cell adhesion and spreading assay. Representative images (20×) of the surface of GelMA hydrogels containing different concentrations (0.1%, 0.075%, 0.05%, and 0.025%) of strontium oxide (SrO). Blue staining indicates cell nuclei; red staining indicates F‐actin filaments (scale bar: 100 *μ*m).

SCAPs cultured on the surface of hydrogels functionalized with MgO (Figure 8), SiO_2_ (Figure 9), and SrO (Figure 10) exhibited cell adhesion, spreading, and an elongated cytoskeletal morphology from Day 1. An increase in cell adhesion and spreading was observed by Day 14, supporting the cytocompatibility of the tested formulations and their ability to sustain cell–material interactions over time.

### 3.4. Morphological and Physicochemical Characterization

#### 3.4.1. Surface Morphology and Topography of Hydrogels

The surface morphological characteristics of the hydrogels are presented in Figure 11a. All formulations exhibited an interconnected porous architecture, with the exception of the GelMA groups functionalized with SrO (0.075% and 0.1%), which displayed a reduced number of surface pores. GelMA hydrogels containing MgO (0.05%) and SiO_2_ (0.025%) exhibited irregular pores in terms of both size and distribution. In contrast, more homogeneous and interconnected pore networks were observed in the groups functionalized with MgO (0.025%) and SiO_2_ (0.05%), which are favorable for supporting cell infiltration, nutrient diffusion, and oxygen exchange.

Figure 11(a) Representative SEM images of the surface and internal structure of control and experimental hydrogels at 250× and 500× magnification (scale bar: 100 *μ*m), (b) bar graph showing the average pore diameter (*μ*m), and (c) porosity percentage (%) of the hydrogels. Bars represent *m* *e* *a* *n* ± *s* *t* *a* *n* *d* *a* *r* *d* *d* *e* *v* *i* *a* *t* *i* *o* *n*. Distinct letters indicate statistically significant differences between groups (one‐way ANOVA/Tukey, *p* < 0.05; *n* = 4).

**Figure:**
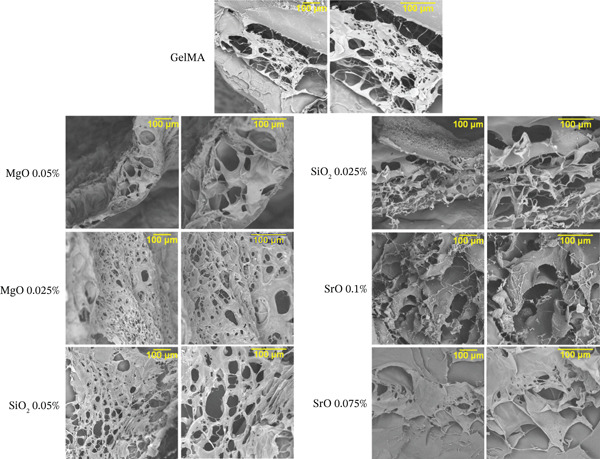
(a)

**Figure:**
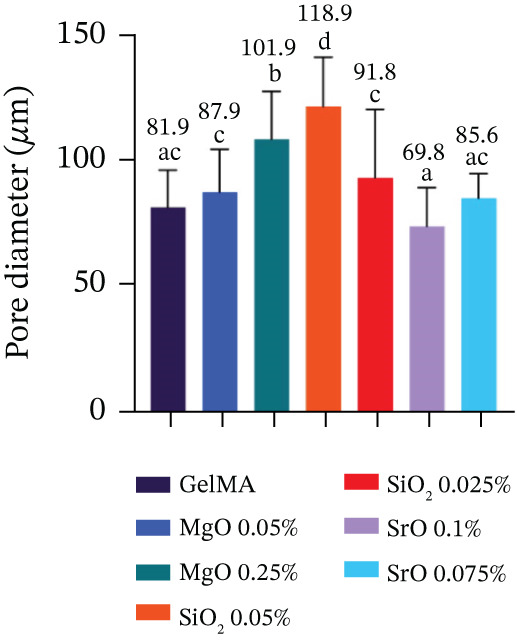
(b)

**Figure:**
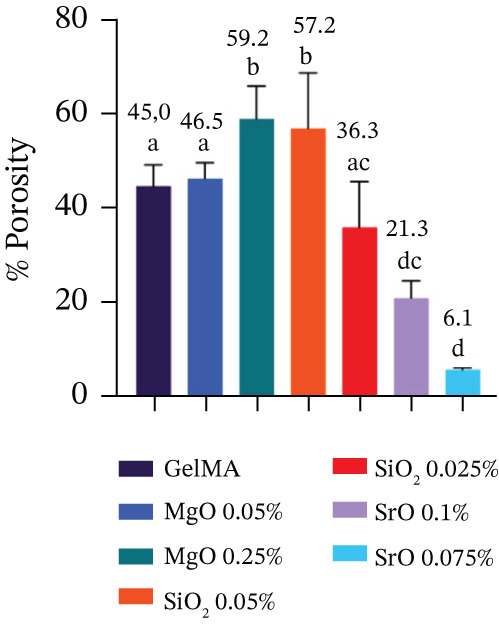
(c)

#### 3.4.2. Porosity and Pore Diameter of GelMA Formulations

Hydrogels functionalized with MgO at 0.025% (101.9 *μ*m) and SiO_2_ at 0.05% (118.9 *μ*m) exhibited significantly larger pore diameters (p < 0.05) compared with the control group (81.9 *μ*m) and the other experimental groups, which ranged from 69.8 to 91.8 *μ*m (Figure 11b). These same formulations also demonstrated the highest porosity values, with 59.2% for MgO at 0.025% and 57.2% for SiO_2_ at 0.05% (Figure 11c). In contrast, hydrogels containing SiO_2_ at 0.025%, SrO at 0.1%, and SrO at 0.075% exhibited markedly lower porosity levels, with values of 36.3%, 21.3%, and 6.1%, respectively.

#### 3.4.3. Swelling Capacity and Degradation Profile of Metal Oxide–Functionalized GelMA Hydrogels

Greater liquid absorption was observed in the control group and in the hydrogels functionalized with MgO (0.025% and 0.05%) during the first 6 h of incubation, with the swelling ratio ranging from 533% to 592% across the different time points. Formulations containing SiO_2_ (0.025% and 0.05%) exhibited increased swelling after 12 h of incubation. The GelMA hydrogels functionalized with SrO (0.075% and 0.1%) remained stable throughout the experimental period. Despite these temporal variations, no statistically significant differences were detected among the groups at any of the evaluated time points (p > 0.05) (Figure 12a).

Figure 12(a) Bar graph showing *m* *e* *a* *n* ± *s* *t* *a* *n* *d* *a* *r* *d* *d* *e* *v* *i* *a* *t* *i* *o* *n* of the swelling ratio of GelMA‐based hydrogels functionalized with different concentrations of MgO (0.05% and 0.025%), SiO_2_ (0.05% and 0.025%), and SrO (0.1% and 0.075%), (b) mass loss over time in the absence of collagenase and, (c) in the presence of collagenase (one‐way ANOVA/Tukey, *p* < 0.05; *n* = 6).

**Figure:**
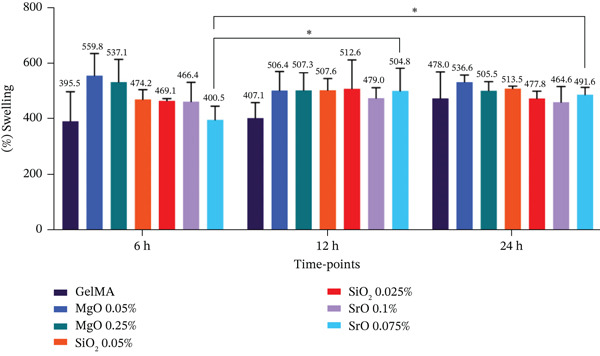
(a)

**Figure:**
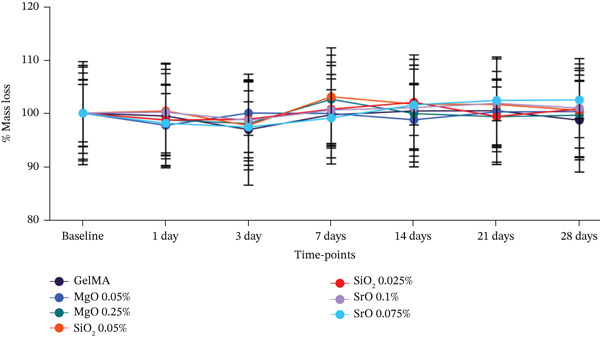
(b)

**Figure:**
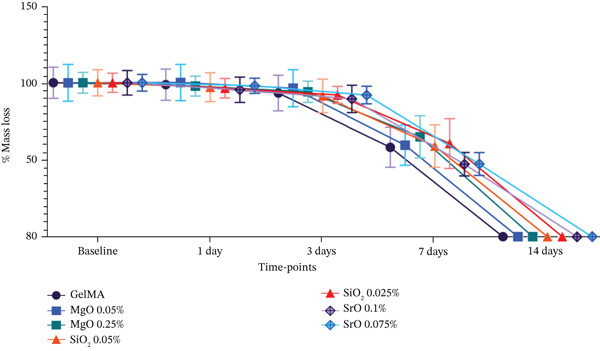
(c)

Degradability analysis revealed that samples incubated in the absence of collagenase exhibited no significant mass loss in either the experimental or control groups, remaining stable over the 28‐day evaluation period. No statistically significant differences were observed among the groups. A slight degradation was noted after 3 days in the GelMA group (4%), as well as in the groups containing SrO (0.075%) and SiO_2_ (0.05%), both showing a 3% mass loss. However, these changes were not significant in terms of final weight, and the samples remained stable throughout the experimental period (Figure 12b). In the presence of collagenase (Figure 12c), all hydrogel formulations showed accelerated degradation beginning on Day 3. This mass loss occurred similarly across all experimental and control groups, with no statistically significant differences detected at any evaluated time point (p > 0.05). Complete degradation of all hydrogels was observed after 2 weeks of incubation.

#### 3.4.4. Sustained Metal Oxide–Derived Species Release From GelMA Formulations

A minimal release of MgO‐derived species was observed during the initial time points (3, 15, 24 hours, and 3 days) for both tested concentrations. No significant difference was detected between the 0.025% and 0.05% MgO‐functionalized groups at Day 7, with both exhibiting concentrations of approximately 0.003 *μ*g/*μ*L on UV‐Vis analysis (Figure 13a). By Day 14, however, an increase in MgO‐derived species release was observed, with slightly higher concentrations detected in the 0.05% group (0.09 *μ*g/*μ*L) compared with the 0.025% group (0.07 *μ*g/*μ*L). A similar release profile was observed for GelMA hydrogels functionalized with SiO_2_ at the early time points. At 7 and 14 days, the hydrogel containing 0.05% SiO_2_ released approximately twice the concentration of SiO_2_‐derived species (0.014 *μ*g/*μ*L) compared with the 0.025% group (0.007 *μ*g/*μ*L) (Figure 13b). Hydrogels functionalized with SrO (0.075% and 0.1%) exhibited a slow and gradual increase in SrO release species profile from 3 h to 7 days, with concentrations ranging from 0.006 to 0.008 *μ*g/*μ*L. A marked increase in SrO release was observed at Day 14, reaching 0.013 *μ*g/*μ*L for the 0.075% group and 0.014 *μ*g/*μ*L for the 0.1% group (Figure 13c). All measured concentrations were above the respective limits of detection (Table S1).

Figure 13Cumulative release of metal oxides incorporated into GelMA hydrogels over time (*n* = 6). (a) Magnesium oxide (MgO) (0.05% and 0.025%), (b) silicon oxide (SiO_2_) (0.05% and 0.025%), and (c) strontium oxide (SrO) (0.1% and 0.075%), expressed in concentration (*μ*g/*μ*L).

**Figure:**
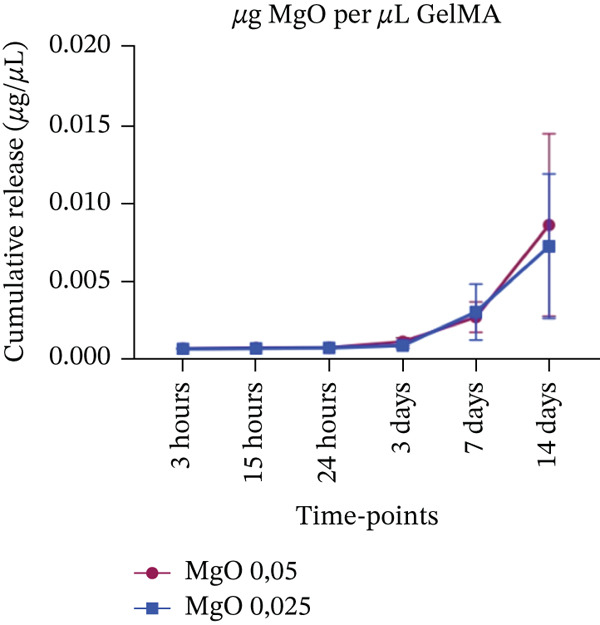
(a)

**Figure:**
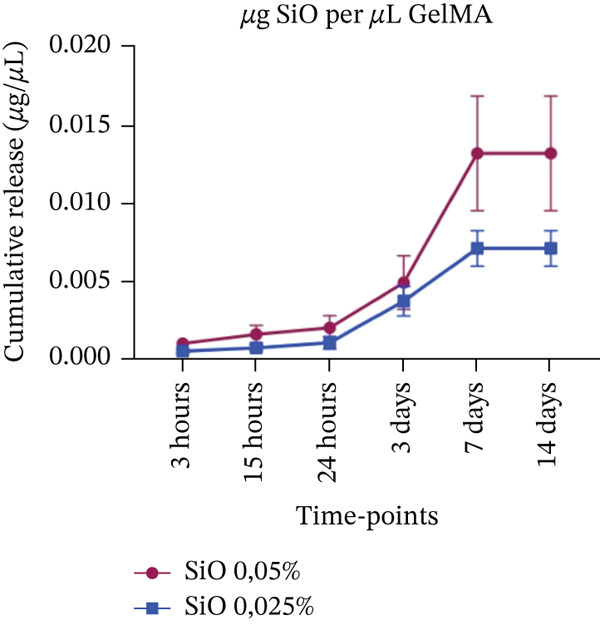
(b)

**Figure:**
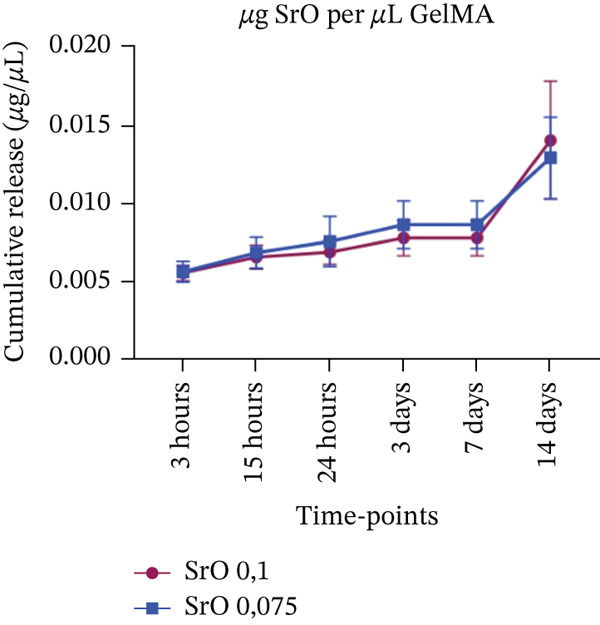
(c)

#### 3.4.5. Chemical Confirmation of Oxide Functionalization in GelMA Hydrogels

The FTIR spectra of the MgO‐containing hydrogels (Figure 14a) exhibit bands in the 550–670 cm^−1^ range, corresponding to the Mg–O stretching vibration. Additional characteristic bands below 1000 cm^−1^ (873, 670, and 580 cm^−1^) are associated with the absorption and complexation of MgO within the hydrogel matrix, which are more pronounced in the formulation containing 0.05% MgO. In the SiO_2_‐functionalized groups (Figure 14b), a weak peak is observed at 1643 cm^−1^, attributed to O–H stretching, and a band at 1094 cm^−1^, corresponding to Si–O–Si stretching vibrations. These signals confirm the partial incorporation of SiO_2_ into the GelMA structure. For the GelMA hydrogels functionalized with SrO (0.075% and 0.1%), the FTIR spectra (Figure 14c) display distinct bands at 733, 810, and 856 cm^−1^, which are indicative of Sr–O bending vibrations, confirming the incorporation of SrO into the hydrogel network.

Figure 14FTIR spectra of GelMA hydrogels functionalized with metal oxides (a) MgO, (b) SiO_2_, and (c) SrO.

**Figure:**
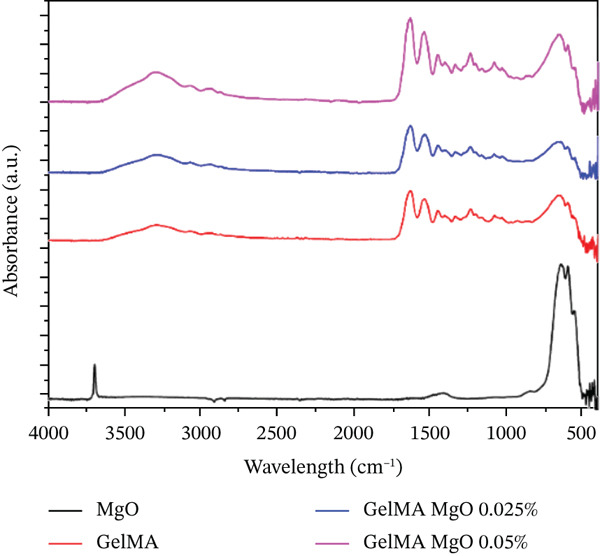
(a)

**Figure:**
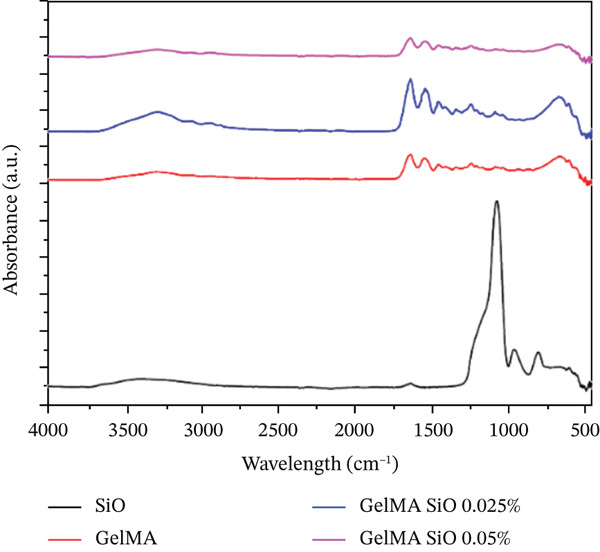
(b)

**Figure:**
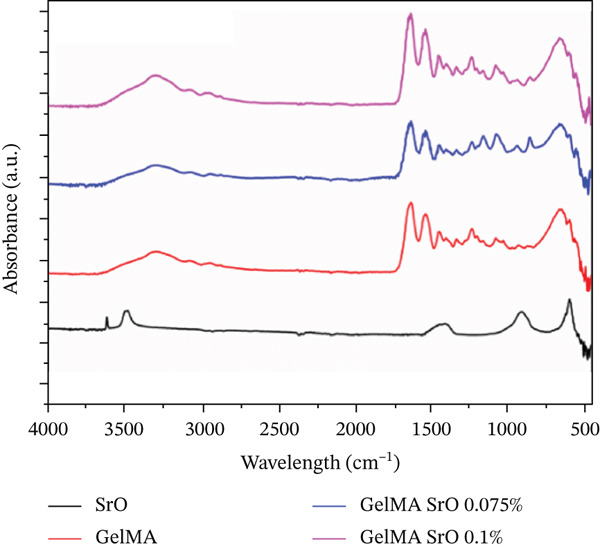
(c)

## 4. Discussion

The dental pulp, a tissue rich in undifferentiated mesenchymal cells, exhibits an intrinsic capacity for tissue repair and regeneration when exposed to the oral environment and mild insults of limited duration [1]. In such cases, minimally invasive and conservative therapies, such as DPC, are recommended to preserve pulp vitality [2]. This procedure involves the application of a material directly onto the exposed pulp to promote the formation of reparative mineralized tissue beneath the defect site [35].

Most capping agents used in these clinical scenarios are considered cytotoxic upon direct contact with pulp tissue due to their alkaline pH, which induces coagulation necrosis and results in the death of numerous undifferentiated mesenchymal cells within the pulp [36, 37]. This response leads to the formation of disorganized and thin tertiary reparative dentin, exhibiting poor tissue‐sealing capacity [3, 38]. Consequently, considerable efforts have been made over the past several decades to develop an ideal material for this clinical application [39–41]. Some researchers have applied the principles of tissue engineering to develop alternative therapeutic materials that are both biocompatible and osteoinductive, thereby facilitating the regeneration and repair of dentin tissue and driving significant advances in regenerative dentistry [42–47].

Recognizing that the development of an ideal material for dentin regeneration remains ongoing, this pursuit motivated the present study, which is aimed at developing and evaluating the efficacy of a novel GelMA hydrogel functionalized with metal oxide nanoparticles for odontogenic differentiation. GelMA is a promising candidate for the regeneration of mineralized tissues such as dentin, as it combines the advantageous properties of both natural and synthetic polymers within a single material [10, 48]. This unique composition enables tailored physicochemical properties, reduces degradability, and allows the biomaterial to persist in the body for extended periods [49].

This characteristic was confirmed in our study, where the mass of the GelMA hydrogel remained stable over 28 days of analysis, irrespective of functionalization with metal oxides. This stability results from the chemical modification of gelatin with methacrylate, which imparts a crosslinked and robust three‐dimensional network to the hydrogel [50]. Conversely, when exposed to a simulated pathological environment containing collagenase, approximately 50% mass degradation of the hydrogel was observed at 7 days, followed by complete degradation at 14 days across all groups evaluated. This behavior is attributed to GelMA matrix metalloproteinase (MMP)–recognizable sites, rendering it susceptible to enzymatic degradation upon collagenase exposure [51].

Collagenase is a proteolytic enzyme commonly employed in in vitro experimental models to simulate the activity of MMPs involved in the degradation of polymeric biomaterials such as GelMA [12, 52]. Its mechanism of action involves cleaving collagen and peptide bonds within GelMA, similarly to MMP‐1, MMP‐3, MMP‐8, and MMP‐13, which are enzymes naturally present in dentin and are primarily activated during pulp inflammation to facilitate reparative dentin formation [53, 54].

In tissue regeneration contexts, biomaterials serve as scaffolding frameworks that facilitate the adhesion, proliferation, and spreading of undifferentiated mesenchymal cells, which are essential processes for promoting cellular differentiation [55, 56]. Therefore, it is critical that these biomaterials exhibit a degradation rate compatible with the deposition of new tissue, ensuring the formation of a homogeneous and consistent mineralized matrix with adequate mechanical properties [57, 58]. Moreover, this controlled and gradual degradation enables the sustained release of bioactive compounds incorporated within the biomaterials at therapeutic concentrations, thereby modulating cellular activity and enhancing osteo/odontogenesis, which ultimately facilitates effective tissue regeneration [59, 60].

In this context, GelMA serves as an excellent carrier for bioactive substances, a property confirmed in our study by the apparent release profiles of metal oxide–derived species assessed by UV‐Vis analysis. The results demonstrated that MgO‐ and SiO_2_‐derived species were released at low concentrations starting from the third day, whereas SrO‐functionalized hydrogels exhibited a controlled release profile beginning as early as 3 h. This sustained release persisted throughout the 14‐day evaluation period for all hydrogel formulations. Although the present methodology does not allow selective quantification of free ionic species, the observed release behavior suggests that the GelMA matrix may modulate the availability of oxide‐derived species over time, potentially limiting their rapid dispersion in the surrounding medium. In this sense, the biomaterial may exert a protective effect on these species, preventing rapid dissolution, which is an important characteristic for maintaining oxide bioavailability in the oral environment. Such controlled availability supports sustained biological activity while minimizing the risk of local cytotoxicity, a balance considered advantageous for dental applications [61, 62].

Previous studies have reported that the incorporation of metal oxide nanoparticles into the composition of scaffolds or hydrogels enhances the bioactivity of undifferentiated mesenchymal cells [10, 63]. This effect was corroborated in our study, where increased cell viability was observed during the early analysis period (7 days), particularly in SCAPs cultured on hydrogels functionalized with SiO_2_ and SrO, compared with the pure GelMA hydrogel. At 14 days, all groups maintained high levels of cell viability; notably, the GelMA hydrogels containing MgO at both concentrations exhibited a threefold increase in viability relative to the earlier time point. Regarding cell morphology, SCAPs displayed an elongated cytoskeleton with filopodial extensions, consistent with an odontoblast‐like phenotype, indicating that the developed biomaterials support cell membrane dynamics, thereby facilitating cell–cell interactions and adhesion to the substrate [64].

This characteristic is essential for activating the cell signaling pathways involved in transduction processes and for promoting cellular differentiation into the desired phenotype [65, 66]. To confirm the effectiveness of the developed formulations in stimulating the odontogenic differentiation of SCAPs, parameters related to ALP activity and mineralized matrix deposition were evaluated. ALP is a key early marker of odontogenic differentiation, playing a crucial role in increasing the concentration of inorganic phosphate and facilitating the formation of hydroxyapatite crystals [5]. These crystals subsequently diffuse into the ECM, where they interact with collagen fibrils to form stable mineralized tissue [67].

The results of our study provide evidence for a contribution of SrO in enhancing ALP activity at 7 days in SCAPs cultivated under osteogenic conditions, particularly at concentrations of 0.075% and 0.05%. Notably, the 0.075% SrO formulation exhibited the highest level of cell differentiation, as evidenced by a statistically significant increase in mineralized matrix deposition at 14 days (232.8%). Supporting our findings, Qi et al. [64] demonstrated that the controlled and sustained release of Sr^2+^ ions from poly (L‐lactide) (PLLA) scaffolds resulted in a biomaterial capable of promoting bone cell adhesion and proliferation, with osteogenic differentiation observed within just 5 days. Furthermore, Zhang et al. [68] reported that Sr^2+^ ions positively regulate the early expression of odontogenesis‐related genes, including Runx2, OPN, and OCN, through activation of the ERK1/2 signaling pathway.

Although the other GelMA hydrogels functionalized with metal oxides did not significantly increase ALP expression at 7 days, the formulations containing 0.05% MgO and 0.025% SiO_2_ stimulated substantial mineralized matrix deposition at 14 days under osteogenic conditions, indicating their potential to promote odontogenic differentiation. In the present study, the positive outcomes related to the differentiation of SCAPs toward an odontogenic phenotype were obtained under culture conditions in which osteogenic medium was employed, a well‐established factor known to promote mineralization [69]. Nevertheless, recent studies by de Souza et al. [70] and Anselmi et al. [71] demonstrated that undifferentiated mesenchymal cells cultured on GelMA‐based hydrogels exhibited enhanced osteogenic and dentinogenic differentiation primarily as a result of biomaterial functionalization with bioactive nanoparticles, irrespective of whether basal or osteogenic culture media were used. These findings indicate that, although osteogenic supplementation contributes to the differentiation process, the bioactive modification of the GelMA matrix plays a central role in modulating lineage‐specific cellular responses, reinforcing the intrinsic potential of nanoparticle‐functionalized hydrogels to stimulate osteogenic and odontoblastic differentiation. Consistent with this interpretation, Chen et al. [63] reported that a chitosan‐based hydrogel incorporating MgO nanoparticles at varying concentrations enhanced osteogenic differentiation in vitro and facilitated bone regeneration in critical‐sized calvarial defects. This phenomenon is attributed to the degradation of MgO into Mg^2+^ and OH^−^ ions, which bind to phosphate groups on the surface and, along with the increased pH in the vicinity of the hydrogel, promote the precipitation of calcium hydroxide (Ca(OH)_2_) and calcium phosphate (CaP), both of which are essential components for dentin mineralization.

Additionally, Mac et al. [72] reported promising outcomes in bone tissue regeneration using mineralized collagen fibers incorporated with SiO_2_ in critical‐sized calvarial defects. The biomaterial facilitated the unidirectional migration of bone cells toward the defect site. Moreover, the presence of silicon resulted in the release of hydrogen gas and water‐soluble silicic acid, molecules known to regulate redox homeostasis and reduce inflammation, as well as to enhance cell proliferation and osteogenic differentiation, thereby positioning this biomaterial as a strong candidate for applications in the regeneration of mineralized tissues.

Studies have shown that the deposition of a mineralized barrier mediated by biomaterials can range from 5 days to 8 weeks, depending on the specific characteristics and composition of the biomaterial [60, 73, 74]. During reparative dentin formation, SrO directly interacts with calcium‐sensing receptors, activating signaling pathways that regulate cell proliferation, ALP expression, and osteogenic differentiation, ultimately leading to the deposition of a robust mineralized matrix [75, 76].

Magnesium plays a crucial role in cellular metabolism by enhancing the proliferation and differentiation of undifferentiated mesenchymal cells into cells exhibiting an osteo/odontoblastic phenotype [77]. It is present at an average concentration of approximately 1% (w/w) within the dentin matrix, with a higher prevalence in regions that have recently undergone mineralization [78]. Both SrO and MgO are trace elements capable of substituting calcium in the hydroxyapatite structure, thereby enhancing dentin′s resistance to acidic environments and mitigating collagen fiber degradation through inhibition of MMP activity [78, 79].

The remineralization potential of SiO_2_ in contact with caries‐affected dentin is well documented in the literature. This nanoparticle infiltrates the dentin collagen matrix, increasing the phosphorus and calcium content, which occupy the interfibrillar spaces and thereby enhance the percentage of mineralized volume formed [80]. Moreover, upon interaction with bone cells, SiO_2_ positively regulates the expression of BMP‐2, a protein involved in the differentiation of undifferentiated mesenchymal cells, promoting bone formation and repair, thus representing an effective strategy for regenerating mineralized tissues [81]. Although the odontogenic properties of ZnO have been extensively reported [82, 83], our study observed significant cytotoxicity of this trace element when exposed to SCAPs under the experimental conditions employed, which precluded the assessment of its potential to induce cell differentiation.

Despite the promising results obtained in this study, it is important to acknowledge that dental pulp tissues subjected to DPC are frequently exposed to inflammatory conditions resulting from bacterial infiltration associated with previous carious lesions [8, 9]. Although low levels of inflammatory mediators are well recognized for their physiological role in initiating dentinogenesis, biomaterials intended for clinical application in such scenarios should ideally also exhibit antimicrobial and anti‐inflammatory properties [46, 71, 84, 85]. These properties are particularly relevant for controlling excessive reactive oxygen species production, which may impair the differentiation capacity of undifferentiated mesenchymal cells and contribute to pain and pulp necrosis [8, 9, 84].

In the present study, inflammatory and antibacterial challenge models were not evaluated, which may limit the direct clinical extrapolation of the findings. Nevertheless, it should be noted that even in vitro and in vivo studies employing simulated inflammatory pulp models have reported inherent limitations in fully reproducing the complexity of the pulp microenvironment and in translating experimental outcomes to clinical scenarios [71, 85]. Despite these constraints, the development of more robust in vitro and in vivo models incorporating inflammatory and antibacterial challenges remains essential to generate more physiologically relevant data and to support the translational assessment of biomaterial performance under clinically relevant conditions.

Additional limitations must also be acknowledged. Although SCAPs were derived from a limited number of donors, the use of a pooled cell population minimized donor‐specific effects and reduced biological variability across experimental conditions. Studies including cells from a larger and more diverse donor pool may further strengthen the generalizability and translational relevance of these findings. Furthermore, future studies should investigate GelMA hydrogels functionalized with bioactive molecules by directly comparing basal and osteogenic culture media to further elucidate biomaterial‐driven effects on cellular differentiation. Overall, this work represents an early‐stage biological and material screening, and additional studies addressing clinically relevant performance parameters will be required prior to translational application.

In addition, the present study did not investigate the molecular mechanisms underlying SCAP responses to metal oxide–functionalized hydrogels. Studies focusing on dentinogenic gene expression and the activation of relevant signaling pathways are therefore warranted to elucidate the molecular basis of the mineralization‐inducing and odontogenic properties of the functionalized hydrogels reported in this study. Finally, as an in vitro investigation, it was not possible to fully replicate the complex biological interactions characteristic of the pulp microenvironment in vivo. Consequently, further research involving animal models and clinical trials is necessary to validate the findings and to elucidate the long‐term efficacy of GelMA hydrogels functionalized with metal oxides under clinical conditions.

## 5. Conclusion

This study demonstrated the successful development of a GelMA hydrogel incorporated with metal oxide nanoparticles (MgO, SiO_2_, and SrO), capable of promoting the proliferation and in vitro differentiation of SCAPs into odontoblast‐like cells. The biomaterials exhibited temporal stability due to the chemical modification of gelatin with methacrylate, which also enabled sustained and controlled release of low concentrations of metal oxides, ensuring their bioavailability and effectiveness in odontogenic differentiation. The formulations containing MgO 0.05%, SiO_2_ 0.025%, and SrO 0.075% showed promising potential to enhance cell proliferation and stimulate odontoblastic differentiation, favoring the deposition of a mineralized matrix barrier under osteogenic conditions. As an early‐stage in vitro investigation, this study provides initial biological and material screening data supporting the bioactivity of metal oxide–functionalized GelMA hydrogels. These findings support their potential for further clinical application as a bioactive material for DPC.

## Funding

This study was supported by the São Paulo Research Foundation (FAPESP) (2020/15971‐5, 2022/05888‐9, 2025/00353‐8) and Coordenação de Aperfeiçoamento de Pessoal de Nível Superior (001). The authors also acknowledge financial support from the Pró‐Reitoria de Pesquisa e Inovação (PRPI)–University of São Paulo (USP), through the support program for new faculty members (Grant No. 22.1.09345.01.2).

## Conflicts of Interest

The authors declare no conflicts of interest.

## Supporting information


**Supporting Information** Additional supporting information can be found online in the Supporting Information section. Figure S1: Calibration curves for each metal oxide used for UV‐Vis analysis of apparent metal oxide release. Table S1: Analytical parameters of UV‐Vis calibration curves used for the apparent release of MgO, SrO, and SiO_2_. Figure S2: Absorbance values of PBS and GelMA + PBS at 600 and 235 nm throughout 14 days.

## Data Availability

The data supporting the findings of this study are available from the authors upon reasonable request.
