# Supplementary material for: Functionalization of Photocrosslinkable GelMA Hydrogel With Metal Oxides for Direct Pulp Capping
Source: Biomed Res Int. 2026 Apr 26;2026:5774660. doi: 10.1155/bmri/5774660 (PMC13110392; doi:10.1155/bmri/5774660)
Supplement: Supplementary file 1 — Supporting Information Additional supporting information can be found online in the Supporting Information section. Figure S1: Calibration curves for each metal oxide used for UV‐Vis analysis of apparent metal oxide release. Table S1: Analytical parameters of UV‐Vis calibration curves used for the apparent release of MgO, SrO, and SiO2. Figure S2: Absorbance values of PBS and GelMA + PBS at 600 and 235 nm throughout 14 days. [file BMRI-2026-5774660-s001.docx]

**Supplemental Material**

**UV–Vis Analysis of Apparent Metal Oxide Release**

Calibration curves were constructed for MgO, SrO, and SiO₂ using known concentrations of each metal oxide dispersed in phosphate-buffered saline (PBS), which was the same medium employed in the release experiments. Absorbance measurements were performed using UV–Vis spectroscopy at the wavelength corresponding to the maximum absorbance of each material. PBS was used as the blank for calibration. The calibration curve is presented on Figure S1.


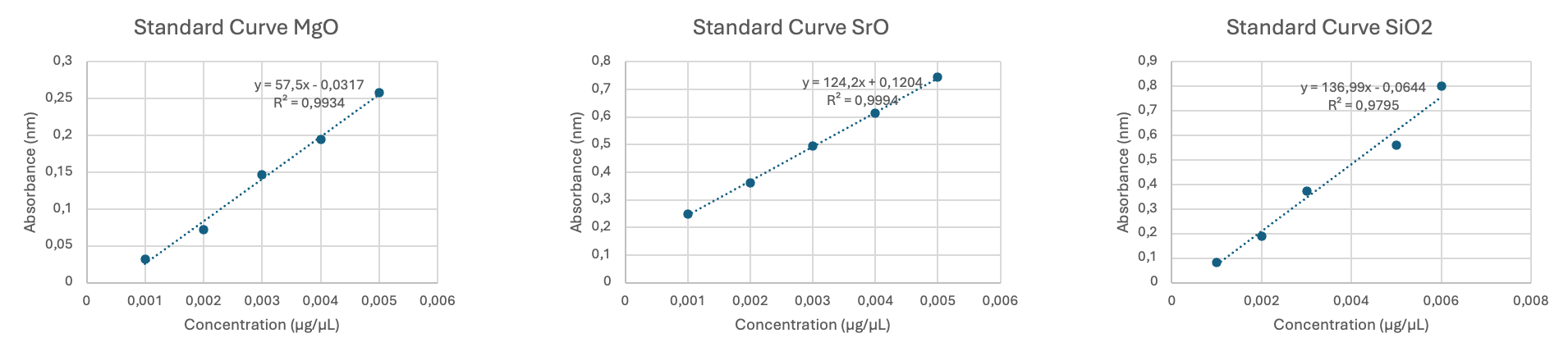


**Figure S1.** Calibration curves for each metal oxide used for UV–Vis Analysis of Apparent Metal Oxide Release.

Apparent concentrations were determined by converting absorbance values to concentration using linear regression of the respective calibration curves. The limits of detection (LOD) and quantification (LOQ) were calculated according to ICH guidelines based on the standard deviation of the background control (σ; n=5) and the slope of the calibration curves (a), using the equations LOD = 3.3σ/a and LOQ = 10σ/a. Table S1 demonstrates all the parameters for each metal oxide.

**Table S1.** Analytical parameters of UV–Vis calibration curves used for the apparent release of MgO, SrO, and SiO₂.

| **Material** | **Slope (a) (Abs/µg·µL⁻¹)** | **R²** | **σ background (Abs)** | **LOD (µg/µL)** | **LOQ (µg/µL)** | **LOD (µg/mL)** | **LOQ (µg/mL)** |
| --- | --- | --- | --- | --- | --- | --- | --- |
| **MgO** | 57.5 | 0.9934 | 0.0015 | 8.6×10⁻⁵ | 2.6×10⁻⁴ | 0.086 | 0.26 |
| **SrO** | 124.2 | 0.9994 | 0.0010 | 2.7×10⁻⁵ | 8.1×10⁻⁵ | 0.027 | 0.081 |
| **SiO_2_** | 136.99 | 0.9795 | 0.0010 | 2.4×10⁻⁵ | 7.3×10⁻⁵ | 0.024 | 0.073 |

**Legend:** Calibration curve slopes (a), coefficients of determination (R²), standard deviation of the background control (σ), and the corresponding limits of detection (LOD) and quantification (LOQ) are shown for each metal oxide. LOD and LOQ were calculated according to ICH guidelines using the equations LOD = 3.3σ/a and LOQ = 10σ/a. The background control consisted of PBS, and all calibration curves were constructed in PBS. The values represent apparent release based on UV–Vis absorbance measurements rather than selective ionic quantification.

To account for matrix-related effects, including potential interference from GelMA-derived components, PBS+GelMA samples were measured independently and used as background controls in the release experiments. Their absorbance values were subtracted from the corresponding sample measurements prior to data analysis. Figure S2 demonstrates that GelMA+PBS samples featured absorbances from day 3.


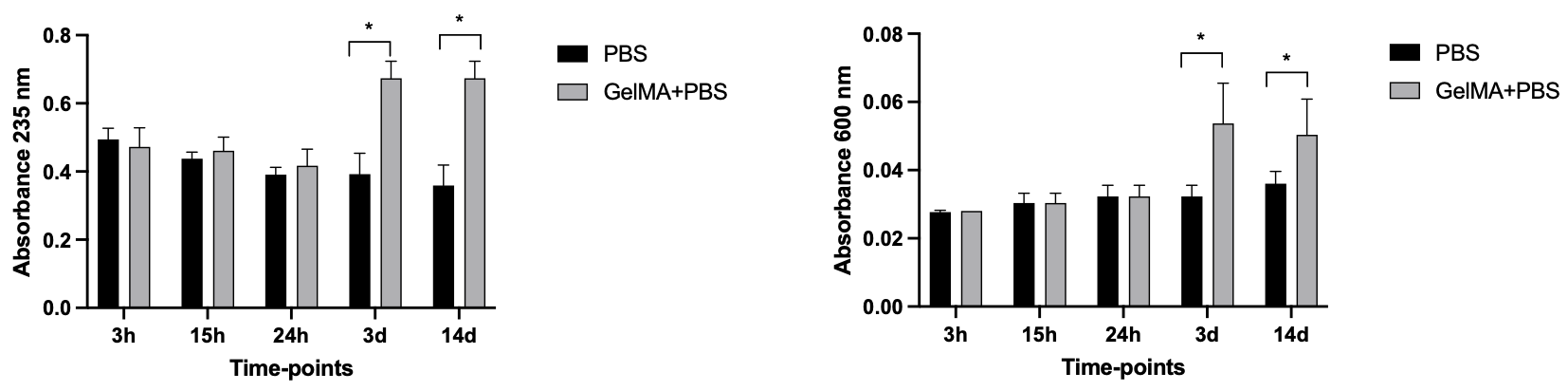


**Figure S2.** Absorbance values of PBS and GelMA+PBS at 600 nm and 235 nm throughout 14 days. Data are mean values (standard deviation). *indicates significant differences (Two-way ANOVA/Tukey’s test; n=5; p<0.05).
